# Point-of-Care Ultrasound of Post-acute COVID-19 Syndrome: A Prospective Cohort Study

**DOI:** 10.7759/cureus.42569

**Published:** 2023-07-27

**Authors:** Elizabeth Dearing, Elizabeth Rempfer, Sarah E Frasure, Hana Akselrod, John E Dobbs, Adrienne N Poon, Juan E Salazar, Dhruvil Prajapati, Keith S Boniface

**Affiliations:** 1 Department of Emergency Medicine, George Washington University School of Medicine and Health Sciences, Washington, USA; 2 Department of Internal Medicine, George Washington University School of Medicine and Health Sciences, Washington, USA; 3 Department of Environmental and Occupational Health, George Washington University School of Medicine and Health Sciences, Washington, USA

**Keywords:** pasc, post-acute covid syndrome, long covid, pocus, covid-19, point-of-care ultrasound, echocardiography, lung ultrasound

## Abstract

Introduction

Acute COVID-19 patients can suffer from chronic symptoms known as post-acute sequelae of SARS-CoV-2 infection (PASC). Point-of-care ultrasound (POCUS) is established in acute COVID, but its utility in PASC is unclear. We sought to determine the incidence of cardiac and pulmonary abnormalities with POCUS in patients with PASC in a COVID-19 recovery clinic.

Methods

This prospective cohort study included adults (>18 years old) presenting with cardiopulmonary symptoms to the COVID-19 recovery clinic. A lung ultrasound and standard bedside echocardiogram were performed by ultrasound-trained physicians. Images were interpreted in real time by the performing sonographer and independently by a blinded ultrasound faculty member. Discrepancies in interpretation were addressed by consensus review. A modified Soldati score was calculated by the sum of the scores in each of the 12 lung zones, with each zone score ranging from 0 to 3 (maximum score of 36). The score was then compared to clinical outcomes and outpatient testing.

Results

Between April and July 2021, 41 patients received POCUS examinations, with 24 of those included in the study. In all, 15 out of 24 (62.5%) had a normal lung ultrasound. Of the nine subjects with lung abnormalities, the median modified Soldati score was 2. Three patients had trivial pericardial effusions, and all had normal left and right ventricular size and function.

Conclusion

The majority (62.5%) of patients presenting to the PASC clinic had a normal pulmonary ultrasound, and the vast majority (87.5%) had normal cardiac ultrasounds. These findings suggest that cardiopulmonary symptoms in PASC may be from etiologies not well evaluated by POCUS.

## Introduction

The novel coronavirus, SARS-CoV-2 (COVID-19), is a respiratory disease with variable acute patient presentations ranging from asymptomatic to acute respiratory distress and death. The cases of COVID-19 as of June 2023 total over 675 million, with over 6.88 million deaths worldwide, leaving millions of people who have recovered from their acute illness [1,2]. The symptoms during and after recovery from the disease are similarly quite variable, with some patients continuing to have symptoms months later, termed post-acute sequelae of SARS-CoV-2 infection (PASC) [3]. A recent study evaluating the health of patients up to two years after the initial COVID-19 infection demonstrated that over half of the survivors suffered long-term sequelae, most commonly fatigue, weakness, and decreased exercise capacity [4]. 

Studies have evaluated patients during hospitalization and after discharge for COVID-19 to monitor clinical changes. A recent study by Alharthy et al. evaluated patients with critical illness acutely during admission and at follow-up for a four-month period after discharge [5]. In all, 11.8% of patients were diagnosed with interstitial lung disease [5]. Another study in patients with moderate to severe acute disease showed that 96.1% had residual computed tomography (CT) findings of ground-glass opacities predominantly in the right lung and, most often, the right lower lobe [6]. However, another study has shown that radiographic testing infrequently reveals pathology to explain these chronic symptoms of COVID-19 infection [7].

Point-of-care ultrasound (POCUS) of the lung has been shown to have excellent sensitivity and specificity for a variety of pulmonary pathology, including pulmonary edema, pleural effusions, and pneumonia. Lung ultrasound has been found to be useful in the diagnosis of acute COVID-19 since the disease preferentially affects the lung periphery [8]. Findings of acute COVID-19 using ultrasound of the lung include pleural thickening, B-lines (alveolar interstitial syndrome), and consolidations with or without air bronchograms. Pleural effusions are an uncommon finding in acute COVID-19 [9,10].

Some studies have specifically followed patients from hospitalization to follow-up with ultrasound. One study found that 31.3% of patients had residual ultrasound findings at three-month follow-up, compared to 79.2% at one-month follow-up [11]. However, this study only included patients who were hospitalized with COVID-19 and, therefore, had prior studies for comparison as well as more severe initial disease.

Patients with symptoms of PASC often have ongoing cardiopulmonary symptoms, such as cough, chest pain, shortness of breath, fatigue, and dyspnea on exertion. In their study, Peghin et al. found that the most frequent PASC symptom was fatigue [12]. While acute radiographic and sonographic findings of COVID-19 have been clearly defined in the literature since early in the COVID-19 pandemic, the same is not true for PASC. Importantly, while POCUS for acute COVID-19 can aid in the initial diagnosis and acute clinical course, as well as monitoring of lung findings during recovery, the value in isolated evaluation for PASC is not known. We sought to evaluate all patients presenting with ongoing cardiopulmonary complaints to the COVID-19 recovery clinic, an outpatient clinic specializing in the evaluation of patients with PASC, for lung and cardiac abnormalities on POCUS.

## Materials and methods

Study design, setting, and population

Identification of Subjects

The study was conducted on patients being treated at an outpatient COVID-19 recovery clinic, George Washington University, Washington, DC, and approved by the institutional review board (IRB NCR202883). This study was part of a larger prospective cohort study designed to understand factors related to PASC. Patients included in this study were adults (>18 years of age) with a prior diagnosis of COVID-19 by positive polymerase chain reaction (PCR) or positive antibody test and a clinical syndrome consistent with COVID-19 and ongoing cardiopulmonary symptoms, including dyspnea, chest pain, pleurisy, hypoxia, hypotension, fatigue, and cough. Patients were excluded if they did not have any ongoing cardiopulmonary symptoms or if they declined to participate. Additional verbal consent for the ultrasound examination was also obtained by both the primary provider and the clinical sonographer for included patients. 

Study protocol

*Sonographers* 

This study included five emergency medicine physicians with ultrasound training: four faculty, each with several years of ultrasound teaching experience in the section of emergency ultrasound, and one ultrasound fellow in the final month of fellowship. Prior to the start of the study, a brief training presentation was provided to each sonographer to define abnormal lung findings, specifically those related to COVID-19, including irregular pleural lines, B-lines, and subpleural consolidations. The training presentation provided definitions and video examples for each specific abnormality option listed on the imaging worksheet. Ultrasounds were performed by one of three ultrasound faculty from the department of emergency medicine or the ultrasound fellow. The fourth ultrasound faculty reviewed these images, blinded to initial interpretation and clinical data.

Ultrasound Equipment

Ultrasounds were obtained using a Butterfly iQ (Butterfly Network, Inc., Guilford, CT) handheld device with the probe connected to an iPhone (Apple, Cupertino, CA) to view images. Images were archived to Butterfly iQ’s online cloud-based Health Insurance Portability and Accountability Act (HIPAA) compliant image archival system. A custom worksheet, created on the Butterfly iQ cloud, was completed for each examination and stored with the images for the study on the cloud (Appendix 1).

*Ultrasound Protocol* 

This prospective cohort study comprised a convenience sample of patients from the COVID-19 recovery clinic from April 2021 to July 2021. Lung and cardiac examinations were performed on patients who met inclusion criteria on days with clinical sonographers present. Six lung zones on each hemithorax were evaluated: superior and inferior zone in anterior (R1 and R2, L1 and L2), lateral (R3 and R4, L3 and L4), and posterior (R5 and R6, L5 and L6) lung fields for a total of 12 lung zones. Patients were supine or sitting for the thoracic exam based on sonographer preference; however, the posterior zones were always obtained with the patient in a sitting position. Video clips were saved from each zone. Focused cardiac exams were also performed on each patient in the supine position, and the standard cardiac views (parasternal long axis, parasternal short axis, apical four-chamber, and subxiphoid) were obtained. E-point septal separation (EPSS) was obtained at the discretion of the sonographer to further evaluate the ejection fraction (normal is 7 mm or less). Tricuspid annular plane systolic excursion (TAPSE) was measured for right heart functional assessment (normal is 17 mm or greater). The IVC was also visualized and measured during change with respiration. These findings were documented immediately after the examination on the electronic worksheet (Appendix 1).

Blinded Interpretation of Images

The initial interpretation of images was performed by the sonographer in real time during the patient evaluation. The sonographer was not blinded to patient demographics or symptoms. The worksheet, including indication for ultrasound, findings, and interpretation of images, was then completed and stored on the Butterfly cloud. The findings for each exam were then transferred to an Excel spreadsheet on a password-protected server. 

The images were independently reviewed by a separate fellowship-trained ultrasound faculty member (SF) from the department of emergency medicine. The reviewer was blinded to clinical information related to the patient visit and the worksheet interpretation completed by the clinician performing the ultrasound.

Consensus Interpretation of Images

Initial or primary interpretation results were compared with the blinded or secondary interpretation results. A consensus review including two of the primary sonographers (ED and KB) and the blinded reviewer (SF) was performed for any findings with disagreement between primary and secondary interpretations in order to resolve disagreements. The consensus reviewers were blinded to a patient presenting symptoms and clinical outcomes. These consensus findings were then used for the final analysis. 

Modified Soldati Scores

The lung abnormalities were scored based on work by Soldati et al. [13]. The original Soldati score is based on 14 lung zones by the addition of a third posterior lung zone bilaterally. Because our standard lung ultrasound protocol includes only two posterior lung fields as opposed to three, our modified Soldati score is based on 12 lung zones: anterior upper and lower, lateral upper and lower, and posterior upper and lower on the left and right sides. Each lung zone was scored as follows:

· Score 0: continuous pleural line with A-lines (i.e., normal lung)

· Score 1: indented pleural line with vertical areas of white arising from the indented pleural line or the pleural-pleural interface and moving with respiration and reaching the end of the screen (B-lines) 

· Score 2: broken pleural line with small to large consolidated areas beneath and white areas below the consolidated lung

· Score 3: dense and largely extended white lung with or without larger consolidations

Each of the 12 lung zones received a score, and the modified Soldati score was the sum of the score of each of these zones for a maximum potential total of 36 for each patient. 

Measurements and data analysis

Medical Record Review

Two additional investigators blinded to the ultrasound results reviewed the medical record and abstracted data using a standard data collection form. Charts were abstracted for the following data: past medical history, medications, prior COVID vaccination, diagnostic testing for COVID, any hospitalization for COVID, any oxygen requirement, prior treatments for COVID (e.g., aspirin and monoclonal antibodies), symptoms at the time of clinic visit, phenotypes present in PASC symptoms, general laboratory testing, and prior imaging. Any discrepancies between the two reviewers were resolved by an additional senior investigator blinded to ultrasound results. Study data were collected and managed using REDCap electronic data capture tools. REDCap is a secure web-based application hosted at BLINDED, which supports data capture and provides an interface for validated data entry, audit trails for tracking data manipulation, and a means of automated exporting [14]. Regular meetings were held between the research assistant and the principal investigator to resolve questions. Best practices in chart review methodology were employed [15].

PASC Phenotype

For the purposes of this study, we categorized patients with PASC into seven phenotypes based on prior literature [3]. 

Cardiac phenotype patients may present with persistent daily chest pain or pressure, documented tachycardia >140 bpm with exertion or resting heart rate >100 bpm, or orthostatic intolerance.

Respiratory phenotype patients may present with daily shortness of breath, daily cough, or oxygenation <95%.

Neurologic phenotype is defined as cognitive impairment with a new need for significant compensatory mechanisms, an inability to complete previous daily tasks due to changes in thinking, the patient performance of two standard deviations below the mean on a cognitive screening battery test, or alteration in taste or smell. 

Vascular phenotype is defined as new hypertension, new hypotension, or syncope. 

Connective tissue/skin phenotype may present with at least two joints with definite synovitis not better explained by another disease, recurrent myalgias severe enough to change behavior, or new recurrent skin rashes post-infection.

Patients may also present with symptoms that are categorized as the miscellaneous phenotype. These symptoms include profound fatigue that limits activities of daily living; documented persistent fever >100.4°F; positive anti-nuclear antibodies, rheumatoid factor, or anti-citrullinated protein antibodies; or elevated erythrocyte sedimentation rate, c-reactive protein, d-dimer, or ferritin.

Statistical Analysis

R Statistical software (R Core Team, v4.1.1) was used to perform all statistical analyses on centralized data from the REDCap database. Descriptive statistics were generated for demographic and comorbidity data. Significance testing was done using the chi-square and Fisher’s exact test with α = 0.05 and by calculating odds ratios with corresponding significance intervals.

## Results

During the study period (April 2021-July 2021), 47 patients presented to the COVID-19 recovery clinic, while ultrasound study team members were present. Six patients were not enrolled because they did not have ongoing cardiopulmonary symptoms or they declined participation, leaving 41 patients enrolled in the ultrasound study. Of the 41 patients, 17 were excluded from the final cohort as they had no positive COVID-19 testing on chart review for a final cohort of 24 patients. Their characteristics are shown in Table 1. Only five patients had a history of asthma, and none had other pre-existing lung disease (e.g., COPD, ILD, and lung cancer). Ultrasounds were performed by four physicians (AD-1, KJ-7, KB-9, and ED-24).

**Table 1:** Baseline characteristics and outcome measures of the study population (n = 24) ^1^n (%); median (IQR) ^2^Fisher's exact test; Wilcoxon rank sum test POCUS, point-of-care ultrasound; PASC, post-acute sequelae of SARS-CoV-2 infection; OHS, obesity hypoventilation syndrome

Characteristic	Overall (n = 24)^1^	Abnormal Soldati score (n = 9)^1^	Normal Soldati score (n = 15)^1^	p-value^2^
Sex				0.19
Female	17 (71%)	8 (89%)	9 (60%)	
Male	7 (29%)	1 (11%)	6 (40%)	
Age	52 (34, 57)	54 (41, 55)	49 (34, 57)	0.61
Race				>0.99
Asian	1 (4.2%)	0 (0%)	1 (6.7%)	
Black or African American	5 (21%)	2 (22%)	3 (20%)	
White	16 (66.7%)	7 (78%)	9 (60%)	
Unknown	2 (8.3%)	0 (0%)	2 (13.3%)	
Time from diagnosis to POCUS				0.54
Short (<3 months)	2 (8.3%)	0 (0%)	2 (13%)	
Intermediate (3 to <6 months)	7 (29%)	2 (22%)	5 (33%)	
Long (≥6 months)	15 (62.5%)	7 (78%)	8 (53%)	
Comorbidities				
Type 2 diabetes	2 (8.3%)	1 (11%)	1 (6.7%)	>0.9
Hypertension	3 (12.5%)	1 (11%)	2 (13%)	>0.9
Hyperlipidemia	5 (21%)	2 (22%)	3 (20%)	>0.9
Coronary artery disease	1 (4.2%)	0 (0%)	1 (6.7%)	>0.9
Heart murmur	1 (4.2%)	1 (11%)	0 (0%)	0.38
Asthma	5 (21%)	2 (22%)	3 (20%)	>0.9
OSA/OHS	7 (29%)	2 (22%)	5 (33%)	0.67
Other cancer	3 (12.5%)	2 (22%)	1 (6.7%)	0.53
Liver disease	1 (4.2%)	0 (0%)	1 (6.7%)	>0.9
Hypothyroidism	2 (8.3%)	1 (11%)	1 (6.7%)	>0.9
Obese	5 (21%)	1 (11%)	4 (27%)	0.61
Stroke	1 (4.2%)	1 (11%)	0 (0%)	0.38
Rheumatoid arthritis	1 (4.2%)	1 (11%)	0 (0%)	0.38
Chronic fatigue	2 (8.3%)	1 (11%)	1 (6.7%)	>0.9
Hospitalization				>0.9
Inpatient floor	1 (4.2%)	0 (0%)	1 (6.7%)	
ICU	1 (4.2%)	0 (0%)	1 (6.7%)	
PASC phenotype (4- to 12-week follow-up)				
Neurologic symptoms/complications	2 (8.3%)	1 (11%)	1 (6.7%)	>0.9
Vascular symptoms/complications	1 (4.2%)	0 (0%)	1 (6.7%)	>0.9
Connective tissue/skin symptoms/complications	2 (8.3%)	0 (0%)	2 (13%)	0.51
Respiratory symptoms/complications	4 (23.5%)	1 (11%)	3 (20%)	>0.9
Cardiac symptoms/complications	2 (8.3%)	0 (0%)	2 (13%)	0.51
Miscellaneous symptoms/complications	1 (4.2%)	0 (0%)	1 (6.7%)	>0.9
PASC phenotype (6- to 12-month follow-up)				
Neurologic symptoms/complications	9 (38%)	3 (33%)	6 (40%)	>0.9
Vascular symptoms/complications	4 (17%)	1 (11%)	3 (20%)	>0.9
Connective tissue/skin symptoms/complications	1 (4.2%)	0 (0%)	1 (6.7%)	>0.9
Respiratory symptoms/complications	4 (17%)	2 (22%)	2 (13%)	0.61
Cardiac symptoms/complications	5 (21%)	3 (33%)	2 (13%)	0.33
Miscellaneous symptoms/complications	8 (33%)	4 (44%)	4 (27%)	0.41

Lung ultrasound

The majority of subjects (15/24, 62.5%) had a completely normal 12-zone lung ultrasound. Of the nine subjects with abnormal lung ultrasounds, the most common finding in at least one lung zone was an irregular or discontinuous pleural line (n = 8, 89%). There were a total of 20 abnormal lung zones out of the 288 total zones for the 24 patients (6.9%). An irregular or discontinuous pleural line focal B-lines was found in 14 of the 20 abnormal zones (70%). Focal B-lines were found in five of the irregular lung zones (25%), while sub-centimeter subpleural consolidations were found in four (20%). Median modified Soldati score was 0 (IQR, 0-1). Of the nine subjects with abnormalities detected on lung ultrasound, the median modified Soldati score was 2 (IQR, 1-3; range, 1-8).

When comparing patients with normal lung POCUS (modified Soldati score of 0) to those with abnormal lung POCUS (modified Soldati score >0), we found no significant difference in the time to diagnosis and ultrasound findings. The time from diagnosis to the clinic visit, which included the ultrasound examination, was divided into short (less than three months), intermediate (three to less than six months), or long (six months or longer). When specifically comparing long to other time periods (short and intermediate combined), there was no statistically significant difference in normal versus abnormal lung findings associated with the time course (Table 2).

**Table 2:** Soldati score compared to time course Time defined from diagnosis to ultrasound examination at the clinic visit and divided into short (less than 3 months), intermediate (3 to <6 months), to long (6 months or longer)

Soldati score classification	Long	Intermediate or short	Odds ratio (95% CI)	p-value
Abnormal Soldati	7	2	3.06 (0.47, 19.88)	0.48
Normal Soldati	8	7		

Abnormal findings in the lung were analyzed by location (anterior, lateral, and posterior) for each side (Table 3). For the anterior lung zones, there were three abnormal findings on the right compared to five on the left. For the lateral lung zones, there were three abnormal findings on the right compared to four on the left. And, in the posterior zones, there were four findings on the right compared to one on the left. When looking at anterior lung zones to lateral lung zones to posterior lung zones, we found no significant difference with findings of eight to seven to five, respectively.

**Table 3:** Abnormal lung zones by location Twenty abnormal lung zones in nine patients

Location	Right	Left	Total
Anterior	3	5	8
Lateral	3	4	7
Posterior	4	1	5
Total	10	10	20

In those patients who received outpatient lung imaging (chest radiography (CXR) or CT), participants with a normal Soldati score most likely had a normal CXR or CT (13/15, 87%). Imaging was at the discretion of the treating physician, including primary care and specialist teams. All imaging obtained after the diagnosis of COVID was considered without specified timing with regards to the acute diagnosis of COVID or the timing of the ultrasound. One of the patients that had an abnormal CT scan showing ground glass opacities had a normal lung ultrasound. However, the CT scan was performed three and a half weeks after the patient’s acute diagnosis of COVID-19. The ultrasound was performed in the clinic three months after the CT scan was obtained and four months after the diagnosis. The other patient with an abnormal CT scan had an ultrasound and CT scan performed on the same day, which was 10 months after the COVID diagnosis. The CT finding that was missed by ultrasound was atelectasis. There were no significant associations between abnormal modified Soldati scores on lung ultrasound and abnormal imaging. None of the participants with abnormal Soldati scores (n = 9) had abnormal chest imaging. 

Finally, the lung ultrasound findings were also analyzed compared to the pulmonary phenotype. Patients with abnormal Soldati scores were more likely to have an absent pulmonary phenotype than a present pulmonary phenotype (n of 6 and n of 3, respectively). Patients with normal Soldati scores were also more likely to have no pulmonary phenotype. Overall, no correlation between lung ultrasound findings and pulmonary phenotype at any time following acute COVID diagnosis (phenotype at four- to 12-week follow-up, p > 0.9; phenotype at six- to 12-month follow-up, p = 0.61) was found (Table 1).

Cardiac ultrasound

All 41 subjects had normal LV function and normal RV size and function. Three subjects were identified to have small pericardial effusions with no evidence of tamponade. Outpatient echocardiography confirmed a small effusion in one patient, pericardial fat pad in a second, and normal in the third. This last patient without a pericardial effusion had the comprehensive echo performed several months prior to POCUS in the clinic. The other two patients had outpatient echocardiograms performed within three weeks of the study echocardiogram.

Consensus review

Any discrepancies in interpretation between the primary and secondary reviewers were reviewed by consensus. This consensus review included two of the primary sonographers in addition to the secondary reviewer. Out of the 288 lung fields evaluated, eight (2.7%) of those had a complete disagreement with the interpretation (e.g., normal by the first reviewer versus focal B-lines by the second reviewer), while 14 (4.9%) had an agreement that there was an abnormality but differed in the final interpretation (e.g., focal B-lines with an irregular/discontinuous pleural line by the first reviewer versus patchy subpleural lesions by the second reviewer). Out of the eight lung fields with complete disagreement, six of those were determined to be normal by consensus, while the other two were found to have an irregular/discontinuous pleural line. Overall, discrepancies found during consensus review usually were the result of the question of B-lines or subtle pleural irregularities (i.e., irregular/discontinuous pleura or thickened pleura). Overall, there was reasonable agreement (92%) between the two reviewers.

## Discussion

In this study of an isolated POCUS exam in adult patients with PASC and ongoing cardiopulmonary symptoms, we found that the majority of patients had normal point-of-care lung and cardiac ultrasounds. Those patients who did have abnormalities had minimal to minor changes overall. These findings are important because POCUS has been used to diagnose the effects of acute COVID-19, and this study shows that this outpatient population with PASC does not have significant pathologic findings that correlate with, nor help to explain, their symptoms. In addition, the length of time from diagnosis of COVID-19 to presentation at the COVID-19 recovery clinic was not found to impact the likelihood of identifying pulmonary or cardiac abnormalities on POCUS.

Of the abnormalities visualized in these patients, most findings were for an irregular or discontinuous pleural line. However, the clinical significance of this is unclear. In addition to pleural line abnormalities, there were a smaller number of subpleural consolidations and areas of focal B-lines. The findings are nonspecific and can be found in a variety of disease states, including pneumonia, pulmonary embolism, and pulmonary contusion, among others. 

There was excellent agreement (92%) between interpretation by the sonographer at the bedside and the asynchronous blinded review. Previous studies have shown a substantial interobserver agreement for normal LUS (=0.79), presence of B-lines (=0.79), and observing three or more B-lines (=0.72) [16]. Less interobserver agreement was found for presence of consolidations, including subpleural consolidation (=0.49), and even less agreement for pleural thickening (=0.23). Another study also showed excellent intraclass correlation coefficients for B-line abnormalities and plural line to consolidation abnormalities; however, the agreement just on pleural line abnormalities was weak (kappa = 0.54) [17]. The defined criteria for the study interpretation provided to sonographers and reviewers included that at least three B-lines must be present in the lung field to qualify as B-lines. Upon consensus review, it was thought that the likely difference between interpretations was a difference in the B-line count (e.g., the primary reviewer counted three, while the secondary reviewer counted two). It is also possible that other comet tail artifacts, such as z-lines, were counted as B-lines by one reviewer and not by another. Still, overall, there was reasonable agreement (92%) between interpretation at the bedside and asynchronous blinded review. 

Since the start of the pandemic, much has been published on acute COVID-19 disease and lung ultrasound. However, less is understood about long-COVID or post-acute COVID-19 syndrome and the utility of isolated cardiac and lung ultrasound in these patients. Many studies have now demonstrated that ultrasound can be a useful tool for monitoring disease progression and improvement, especially in hospitalized patients and upon follow-up. These studies show that, as expected, lung ultrasound findings improve over time [18]. A recent study also used ultrasound to monitor the evolution of severe COVID pneumonia in ICU patients after hospital discharge [5]. Overall, there seems to be a good consensus that ultrasound is a useful tool for the diagnosis of acute COVID-19 as well as monitoring of disease progression in patients with lung involvement. Our study, in contrast, evaluates outpatients with resolved acute COVID-19 but persistent cardiopulmonary symptoms consistent with PASC and seeks to characterize lung and cardiac ultrasound findings present in this population. 

Based on these studies and the documented improvement in ultrasound over time, it is not surprising that there was a relatively low yield of abnormal findings in our patients, given the time from disease onset to POCUS examination in a relatively healthy population (with only one reported hospitalization in the cohort). Our study utilizes ultrasound in an outpatient setting for all patients regardless of prior hospitalization or imaging with ongoing cardiopulmonary symptoms after a documented COVID-19 infection. This study used an isolated POCUS examination, which may be more generalizable to all patients presenting with PASC since most patients with COVID-19 are not hospitalized. Another strength of this study is that the subjects were all patients with documented COVID-19 infection by laboratory testing who presented with persistent cardiopulmonary symptoms. 

The use of POCUS for cardiac and lung evaluation is beneficial to the study as it is without ionizing radiation and is an easily reproducible and accessible imaging study. Additionally, Altersberger et al. recently published a review document advocating for the use of lung and cardiac ultrasound in PASC patients. They note that while more literature is needed in the context of the post-COVID syndrome to determine which patients benefit the most from ultrasound examination, lung ultrasound and echocardiography hold great potential [19]. The differential diagnosis for cardiopulmonary symptoms in this patient population includes diseases that can produce abnormalities detected by ultrasound. POCUS is rapid and easy to perform at the bedside and can exclude some of these diseases, although the yield was low in this cohort. 

There are several limitations to this study. This was an observational study of all patients presenting to an outpatient COVID recovery clinic. The COVID-19 recovery clinic began seeing patients at a point early in the pandemic when testing was not widely available. Therefore, a positive COVID-19 test result was not required to be seen in the clinic if a clinical diagnosis was made based on symptoms. As a result, some patients did not have proof of positive COVID-19 test results (antigen, PCR, or antibody) documenting past infection. Because we only wanted to include patients in our study with known COVID-19, we excluded anyone that did not have a documented positive result in the electronic health record. Additionally, as the COVID-19 recovery clinic draws its patients from a large catchment area, some clinic patients came from surrounding health systems, which limited the capacity for chart review. We chose to limit the patients in our study to those with definitive evidence of past COVID-19 testing, leading to a small number of patients in the study. This small sample size may have caused some important differences to be missed.

There was no defined time interval between initial diagnosis and further testing, including POCUS examination, with patients presenting to the clinic at variable times after their initial diagnosis with some presenting within a few months and some presenting over a year after initial symptoms. Furthermore, other outpatient testing, such as CXR, CT, or pulmonary function testing, was not always performed. If performed, there was no set time for this testing from the time of initial diagnosis or from the POCUS examination. We found that some patients received more acute testing during the short (less than three months) follow-up period, while others received testing only during the long (greater than six months) follow-up period. 

Patients in this study were noted to be relatively young, with a mean age of 47 years, and with minimal co-morbidities. We hypothesize that patients with significant co-morbidities may be following with specialists (e.g., cardiology and pulmonology) as opposed to the COVID-19 clinic. Therefore, these findings may not be as generalizable to all patients with PASC, especially older patients with pre-existing cardiopulmonary disease. This patient population may be a higher yield population for cardiopulmonary ultrasound.

As the pandemic has continued, additional variants of COVID-19 have emerged. Our study took place during April to July 2021 in Washington, DC, and prior to the delta and omicron variants. Therefore, it is not known if these results would differ when looking at patients with newer variants of the disease. The current omicron variants have anecdotally led to less severe illness overall and, therefore, presumably, may also lead to fewer pulmonary findings both acutely and in PASC. Furthermore, because this study was performed in early to mid-2021, many of the patients presenting would not have had the opportunity to receive a full vaccination series. It is also unclear what impact vaccination would have had on our study.

Lastly, the imaging technology chosen for the POCUS exams may have impacted the results of the study. We chose the Butterfly iQ system using the iPhone display for image acquisition and real-time interpretation. This may have resulted in lower resolution than larger cart-based systems, given the hand-held technology and the smaller screen size. The images were reviewed on a laptop for blinded review and the consensus review of any discordant examinations.

## Conclusions

In our study, there was excellent agreement (92%) between interpretation by the sonographer at the bedside and the asynchronous blinded review. The majority (62.5%) of patients presenting to a dedicated clinic for the care of post-acute COVID-19 with ongoing cardiopulmonary symptoms have a normal pulmonary ultrasound. Furthermore, the vast majority (87.5%) had a normal cardiac ultrasound, with trace/trivial pericardial fluid being the only cardiac abnormality found. Overall, these findings suggest that cardiopulmonary symptoms in PASC may be from etiologies not well evaluated by POCUS. Future studies should work to identify a PASC cohort where POCUS may be a higher yield.

## Appendices

Appendix 1 

**Table 4:** COVID recovery clinic lung and cardiac ultrasound worksheet Worksheet completed by the physician performing ultrasounds in real-time IVC, inferior vena cava

Lung and cardiac ultrasound worksheet	
Indication(s) for exam	
❑	Dyspnea
❑	Chest pain
❑	Pleurisy
❑	Hypoxia
❑	Hypotension
❑	Chronic fatigue
❑	Cough
❑	Other:
Views obtained with adequate views		
❑	R1 - right anterior/superior thorax	
❑	R2 - right anterior/inferior thorax
❑	R3 - right lateral/superior thorax
❑	R4 - right lateral/inferior thorax
❑	R5 - right posterior/superior thorax
❑	R6 - right posterior/inferior thorax
❑	L1 - left anterior/superior thorax
❑	L2 - left anterior/inferior thorax
❑	L3 - left lateral/superior thorax
❑	L4 - left lateral/inferior thorax
❑	L5 - left posterior/superior thorax
❑	L6 - left posterior/inferior thorax
❑	Parasternal long axis
❑	Parasternal short axis
❑	Apical 4 chamber
❑	Subxiphoid
❑	IVC
Thoracic findings	
	R1
❑	Focal B-lines
❑	Diffuse B-lines
❑	Confluent B-lines
❑	Normal lung sliding
❑	Normal pleural line
❑	Irregular/discontinuous pleural line
❑	Thickened pleural line
❑	Subpleural lesions - patchy
❑	Subpleural lesions - strip
❑	Subpleural lesions - sub-centimeter consolidation
❑	Air bronchograms
❑	Consolidation >1 cm
❑	Pleural effusion - small
❑	Pleural effusion - medium
❑	Pleural effusion - large
	R2
❑	Focal B-lines
❑	Diffuse B-lines
❑	Confluent B-lines
❑	Normal lung sliding
❑	Normal pleural line
❑	Irregular/discontinuous pleural line
❑	Thickened pleural line
❑	Subpleural lesions - patchy
❑	Subpleural lesions - strip
❑	Subpleural lesions - sub-centimeter consolidation
❑	Air bronchograms
❑	Consolidation >1 cm
❑	Pleural effusion - small
❑	Pleural effusion - medium
❑	Pleural effusion - large
	R3
❑	Focal B-lines
❑	Diffuse B-lines
❑	Confluent B-lines
❑	Normal lung sliding
❑	Normal pleural line
❑	Irregular/discontinuous pleural line
❑	Thickened pleural line
❑	Subpleural lesions - patchy
❑	Subpleural lesions - strip
❑	Subpleural lesions - sub-centimeter consolidation
❑	Air bronchograms
❑	Consolidation >1 cm
❑	Pleural effusion - small
❑	Pleural effusion - medium
❑	Pleural effusion - large
	R4
❑	Focal B-lines
❑	Diffuse B-lines
❑	Confluent B-lines
❑	Normal lung sliding
❑	Normal pleural line
❑	Irregular/discontinuous pleural line
❑	Thickened pleural line
❑	Subpleural lesions - patchy
❑	Subpleural lesions - strip
❑	Subpleural lesions - sub-centimeter consolidation
❑	Air bronchograms
❑	Consolidation >1 cm
❑	Pleural effusion - small
❑	Pleural effusion - medium
❑	Pleural effusion - large
	R5
❑	Focal B-lines
❑	Diffuse B-lines
❑	Confluent B-lines
❑	Normal lung sliding
❑	Normal pleural line
❑	Irregular/discontinuous pleural line
❑	Thickened pleural line
❑	Subpleural lesions - patchy
❑	Subpleural lesions - strip
❑	Subpleural lesions - sub-centimeter consolidation
❑	Air bronchograms
❑	Consolidation >1 cm
❑	Pleural effusion - small
❑	Pleural effusion - medium
❑	Pleural effusion - large
	R6
❑	Focal B-lines
❑	Diffuse B-lines
❑	Confluent B-lines
❑	Normal lung sliding
❑	Normal pleural line
❑	Irregular/discontinuous pleural line
❑	Thickened pleural line
❑	Subpleural lesions - patchy
❑	Subpleural lesions - strip
❑	Subpleural lesions - sub-centimeter consolidation
❑	Air bronchograms
❑	Consolidation >1 cm
❑	Pleural effusion - small
❑	Pleural effusion - medium
❑	Pleural effusion - large
	Other right thorax findings:
	L1
❑	Focal B-lines
❑	Diffuse B-lines
❑	Confluent B-lines
❑	Normal lung sliding
❑	Normal pleural line
❑	Irregular/discontinuous pleural line
❑	Thickened pleural line
❑	Subpleural lesions - patchy
❑	Subpleural lesions - strip
❑	Subpleural lesions - sub-centimeter consolidation
❑	Air bronchograms
❑	Consolidation >1 cm
❑	Pleural effusion - small
❑	Pleural effusion - medium
❑	Pleural effusion - large
	L2
❑	Focal B-lines
❑	Diffuse B-lines
❑	Confluent B-lines
❑	Normal lung sliding
❑	Normal pleural line
❑	Irregular/discontinuous pleural line
❑	Thickened pleural line
❑	Subpleural lesions - patchy
❑	Subpleural lesions - strip
❑	Subpleural lesions - sub-centimeter consolidation
❑	Air bronchograms
❑	Consolidation >1 cm
❑	Pleural effusion - small
❑	Pleural effusion - medium
❑	Pleural effusion - large
	L3
❑	Focal B-lines
❑	Diffuse B-lines
❑	Confluent B-lines
❑	Normal lung sliding
❑	Normal pleural line
❑	Irregular/discontinuous pleural line
❑	Thickened pleural line
❑	Subpleural lesions - patchy
❑	Subpleural lesions - strip
❑	Subpleural lesions - sub-centimeter consolidation
❑	Air bronchograms
❑	Consolidation >1 cm
❑	Pleural effusion - small
❑	Pleural effusion - medium
❑	Pleural effusion - large
	L4
❑	Focal B-lines
❑	Diffuse B-lines
❑	Confluent B-lines
❑	Normal lung sliding
❑	Normal pleural line
❑	Irregular/discontinuous pleural line
❑	Thickened pleural line
❑	Subpleural lesions - patchy
❑	Subpleural lesions - strip
❑	Subpleural lesions - sub-centimeter consolidation
❑	Air bronchograms
❑	Consolidation >1 cm
❑	Pleural effusion - small
❑	Pleural effusion - medium
❑	Pleural effusion - large
	L5
❑	Focal B-lines
❑	Diffuse B-lines
❑	Confluent B-lines
❑	Normal lung sliding
❑	Normal pleural line
❑	Irregular/discontinuous pleural line
❑	Thickened pleural line
❑	Subpleural lesions - patchy
❑	Subpleural lesions - strip
❑	Subpleural lesions - sub-centimeter consolidation
❑	Air bronchograms
❑	Consolidation >1 cm
❑	Pleural effusion - small
❑	Pleural effusion - medium
❑	Pleural effusion - large
	L6
❑	Focal B-lines
❑	Diffuse B-lines
❑	Confluent B-lines
❑	Normal lung sliding
❑	Normal pleural line
❑	Irregular/discontinuous pleural line
❑	Thickened pleural line
❑	Subpleural lesions - patchy
❑	Subpleural lesions - strip
❑	Subpleural lesions - sub-centimeter consolidation
❑	Air bronchograms
❑	Consolidation >1 cm
❑	Pleural effusion - small
❑	Pleural effusion - medium
❑	Pleural effusion - large
	Other left thorax findings:
Echo findings	
Left ventricular function		
❑	Normal (>50%)
❑	Depressed (30-50%)
❑	Severely depressed (<30%)
Right ventricular function	
❑	Normal
❑	Septal flattening/dyskinesis
❑	Right ventricular dilation
❑	TAPSE >1.7 cm
❑	McConnell's sign
Pericardium	
❑	No pericardial effusion
❑	Pericardial effusion - small
❑	Pericardial effusion - medium
❑	Pericardial effusion - large
❑	Pericardial effusion with signs of tamponade/R-sided collapse
IVC	
❑	DMax < 2.1 cm
❑	DMax ≥ 2.1 cm
❑	Collapsibility index >50%
❑	Collapsibility index <50%
Interpretation	
Thoracic ultrasound	
❑	No sonographic evidence of acute pulmonary disease	
❑	Right pneumothorax
❑	Left pneumothorax
❑	Bilateral pneumothorax
❑	Right pleural effusion
❑	Left pleural effusion
❑	Bilateral pleural effusion
❑	Alveolar interstitial syndrome (diffuse)
❑	Right lung consolidation
❑	Left lung consolidation
❑	Bilateral consolidation
❑	Right pleural thickening/irregularity
❑	Left pleural thickening/irregularity
❑	Right subpleural lesion
❑	Left subpleural lesion
❑	Alveolar interstitial syndrome (diffuse/bilateral)
❑	Right alveolar interstitial syndrome (focal)
❑	Left alveolar interstitial syndrome (focal)
Cardiac ultrasound	
❑	No sonographic evidence of active cardiac disease	
❑	LV - depressed LVEF (30-50%)
❑	LV - severely depressed LVEF (<30%)
❑	RV - evidence of right heart strain
❑	Pericardial effusion
❑	Pericardial tamponade
❑	RV - evidence of right heart strain
